# Identification of Coumarins and Antimicrobial Potential of Ethanolic Extracts of *Dipteryx odorata* and *Dipteryx punctata*

**DOI:** 10.3390/molecules27185837

**Published:** 2022-09-08

**Authors:** Bruna Cristine Martins de Sousa, Santana Pinto de Castro, Katiane Araújo Lourido, Aline Aparecida München Kasper, Geomarcos da Silva Paulino, Camila Delarmelina, Marta Cristina Teixeira Duarte, Adilson Sartoratto, Thiago Almeida Vieira, Denise Castro Lustosa, Lauro Euclides Soares Barata

**Affiliations:** 1 Instituto de Biodiversidade e Florestas (IBEF), Universidade Federal do Oeste do Pará (UFOPA), Santarem 68040-255, Brazil; 2 Programa de Pós-Graduação em Sociedade, Natureza e Desenvolvimento (PPGSND), Universidade Federal do Oeste do Pará (UFOPA), Santarem 68040-255, Brazil; 3 Rede Integrada de Desenvolvimento Humano (RIDH), Universidade Federal do Oeste do Pará (UFOPA), Santarem 68040-255, Brazil; 4 Centro Pluridisciplinar de Pesquisas Químicas, Biológicas e Agrícolas, Universidade Estadual de Campinas, Campinas 13148-218, Brazil

**Keywords:** antimicrobial activity, chromatography, cumaru extracts, natural products

## Abstract

*Dipteryx odorata* and *Dipteryx punctata* are species native to the Amazonian, traded by extractivists to obtain coumarin. We aimed to analyze the presence of coumarin in the ethanolic extracts of leaves, branches and fruits of *D. odorata* and *D. punctata* and to evaluate the antimicrobial activity of these extracts against phytopathogenic fungi and bacteria of clinical interest. Chemical analyses were performed by thin layer chromatography (TLC) and by gas chromatography coupled to mass spectrometry (GC-MS). For the antifungal assays, the fungi used were *Cercospora longissima*, *Colletotrichum gloeosporioides*, two isolates of *Fusarium* spp. and *Sclerotium rolfsii*, and the antibacterial assay was performed using the minimum inhibitory concentration (MIC) test with *Burkholderia cepacia*, *Escherichia coli*, *Pseudomonas aeruginosa* and *Staphylococcus aureus* bacteria. In *D. odorata* seed extracts and in *D. punctata* husks, endocarps, and seeds, we identified 1,2-benzopyrone. *D. odorata* endocarp extracts and *D. punctata* seeds provided the greatest decrease in mycelial growth of the evaluated phytopathogens, showing promise as an alternative control. The husk and endocarp extracts of both species had a weak effect on *E. coli*. This research is the first to compare the different parts of species of the genus *Dipteryx* and to evaluate the use of husks and endocarps of *D. punctata* fruits to obtain coumarin. Chemical analyses used to quantify the compounds existing in the extracts, and tests with phytopathogens in vitro and in vivo are currently being carried out.

## 1. Introduction

The genus *Dipteryx*, belonging to the Fabaceae family, includes neotropical plant species, native to Central and South American countries [1]. Among these species, *Dipteryx odorata*, known as cumaru or tonka bean, stands out in the Amazon for its timber potential, seed commercialization and phytotherapic use. Coumarin (1,2-benzopyrone) can be extracted from the seeds of *Dipteryx odorata*, being widely used in the perfumery and cosmetics industries as an essence fixer. However, coumarins present such significant structural diversity that their biological activities are the most varied [2]. Tests to identify the action of these compounds are important, as they organize information about species that have bioactive natural compounds. Other species, such as *Dipteryx punctata*, lack information about their chemical aspects and biological activities, thus becoming important sources for studies.

Natural products, such as plant extracts, have been used since the beginning of human history [3]. As they are biodegradable, renewable and contain potentially active substances, they have aroused interest in the discovery of various microbiological activities [4]. In this sense, natural molecules with antifungal properties have been a focus of research in recent years [5], highlighting the use of products of natural origin to control plant diseases, as an alternative to the indiscriminate use of fungicides. In various industries, these products are part of the development of future biocompatibles, either as an in natura product/component or as a model for chemical synthesis or semi-synthesis of products with necessary fungitoxic characteristics [6].

Some fungi that affect crops of economic interest are *Cercospora longissima* (Cugini) Sacc., the causal agent of *Cercospora* leaf spot on lettuce [7]. *Colletotrichum* spp. are also associated with losses in vegetables and other crops [8]. The *Fusarium* genus is mainly composed of soil fungi, with a cosmopolitan distribution and active in the decomposition of plant cell substrates, some of which cause plant diseases [9], such as the species *F. graminearum*, *F. moniliforme*, *F. oxysporum* and *F. verticillioides*, known to infect cereal, fruit, vegetable crops, among others [10]. *Sclerotium rolfsii* is a soil polyphagous phytopathogenic fungus that causes significant crop yield losses worldwide, particularly in tropical and subtropical countries [11]. Chemical control in many cases is not so efficient, as the fungus most often penetrates the vascular tissue of the plant; in addition, its use causes numerous harmful effects on man and nature [12], which is important in the study of control based on plant products.

Aqueous extracts of garlic (*Allium sativum*), rue (*Ruta graveolens*), cinnamon (*Cinnamomum verum*), clove (*Syzygium aromaticum*), horsetail (*Equisetum arvense*), eucalyptus (*Eucalyptus glubulus*), mint (*Mentha* sp.), jabuticaba (*Plinia cauliflora*), São Caetano melon (*Momordica charantia*) and Indian neem (*Azadirachta indica*) were tested on the fungi *Aspergillus* sp., *Penicillium* sp., *Cercospora kikuchii*, *Colletotrichum* sp., *Fusarium solani* and *Phomopsis* sp., and action was identified for clove, garlic and cinnamon extracts at a concentration of 20% [13]. Twenty plant extracts were evaluated regarding the control of mycelial growth of *C. gloeosporioides* and only those of oleander (*Nerium oleander*), eucalyptus (*Eucalyptus citriodora*) and São Caetano melon (*Momordica charantia*) caused inhibition of more than 50% of the mycelial growth of this phytopathogen [14].

In pharmacology, natural extractives have been investigated in the search for substances with antimicrobial potential, mainly in the study of species that are used in traditional medicine, such as the genus *Dipteryx*. In addition, the increase in diseases caused by microorganisms, adverse effects [15], more severe microbial infections in immunodeficient patients, requiring alternatives to improve multidrug resistance [16], and infections resistant to existing antibiotics have stimulated the search for effective and less toxic substances [17,18]. Some widely studied microorganisms are the *Burkholderia cepacia* complex (Bcc), opportunistic pathogens acquired by patients with cystic fibrosis (CF) in later years [19]; *Escherichia coli*, Gram-negative bacteria classified by the World Health organization (WHO) as high priority pathogens [20]; *Pseudomonas aeruginosa*, which is also a Gram-negative, opportunistic bacterium responsible for serious nosocomial infections with high rates of mortality and morbidity [21]. In addition, there is an urgent need to develop alternatives or control infections caused by *Staphylococcus aureus* [22].

In this context, the objective of this study was to analyze the presence of coumarin in the ethanolic extracts of leaves, branches and fruits of *D. odorata* and *D. punctata,* and to evaluate the antimicrobial activity of these extracts against phytopathogenic fungi and bacteria of clinical interest.

## 2. Results

For the two plant species, the highest yields were obtained from extracts from the husks and seeds of the fruits, which did not differ from each other. The lowest yields were observed for extracts of *D. odorata* endocarps and *D. punctata* endocarps and leaves (Table 1).

In the verification of the presence of coumarin (1,2-benzopyrone) in the extracts, using the TLC method, we found coumarin in the seeds of *D. odorata* and in the seeds, endocarps and husks of *D. punctata*, visualized in the length of waveform of 365 nm, with a retention factor of 0.5 to 0.6 when compared to synthetic 1,2-benzopyrone.

The analyses by gas chromatography coupled to mass spectrometry showed (GS-MS) variations in the chemical composition of the extracts of the two plant species under study. For *D. odorata* branch extracts, taraxasterol (RT of 49.39 min) was the major constituent (63.9%). This compound belongs to the class of triterpenes. In the husks of *D. odorata* fruits, spathulenol, caryophyllene oxide, α-caryophyllene, phenanthrene [3,2*b*]furan-4-methanol-1,2,3,4,4a,5,6,6a,7,11,11a,11b, dodecahydro-4,7,11-trimethyl and germacrene D were identified. In the endocarp extract, a greater number of substances was identified, with six compounds (aromadendrene, β-cubebene, caryophyllene, δ-cadinene, δ-muurolene and copaene) that were not observed in the extracts from leaves, branches and husks. The presence of coumarin was confirmed in the seeds, found by the thin layer chromatography (TLC) analysis (Table 2).

In the phytochemical analysis of *D. punctata* extracts, the major constituent of the leaves was taraxasterol (51.9%), which was also observed in the extract of the branches (15.1%), with a retention time (RT) of 49.39 min. In the extracts of *D. punctata* fruit husks, among the identified compounds, spathulenol (14.6%), ethyl ester of oleic acid (9.4%) and phenanthro [3,2b]furan-4-methanol 1,2,3,4,4a,5,6,6a,7,11,11a,11b,dodecahydro-4,7,11-trimethyl (5.7%) had the highest relative percentage. The presence of coumarin in the extracts of the husks, endocarps and seeds of *D. punctata* was indicated in the TLC and confirmed by GC-MS and, only in the extracts of the seeds, was found in addition to coumarin, another compound that belongs to the class of coumarins, 3,4-dihydrocoumarin, with a relative percentage of 25.2% (Table 3).

For alternative control of phytopathogens using *D. odorata* extracts, there was a significant difference for both the isolated factors (concentrations, extracts and phytopathogens) and for the interaction between them. The extract obtained from the leaves, at a concentration of 30%, and all concentrations of the extract obtained from the endocarps, with the exception of 10%, caused the greatest reductions in the mycelial growth of *Cercospora longissima*, differing from the other treatments. The bark extract provided an increase in the growth of this fungus at all concentrations tested (Table 4). The reduction in the diameter of *C. longissima* colonies was 30.8% for the leaf extract and ranged from 28.2% to 38.5% for the endocarp extract, when compared to the control.

For *Colletotrichum gloeosporioides* and *Fusarium* spp., the endocarp extract, at concentrations of 40% and 50%, provided the greatest reductions in the average diameter of their colonies (Table 4). *C. gloeosporioides* showed a reduction of 64.6% and the isolates of *Fusarium* spp. showed a reduction of 54.7% (lettuce) and 59.5% (kale), in relation to the control. The lowest mycelial growth of *Sclerotium rolfsii* was observed in the concentration of 10% of the seed extract (Table 4), with a reduction of 34.6% in the diameter of the colonies. Regarding coumarin (1,2-benzopyrone), there was a reduction in the mycelial growth of the fungi *C. gloeosporioides* and *Fusarium* spp. (lettuce and kale), when compared to the control (Table 4). However, this treatment was not superior to any of the results obtained for the extracts that reduced fungal growth.

In the analysis of *D. punctata*, the concentrations of 10% and 30% of the seed extract provided the smallest diameter means for *C. longissima* (Table 5). In the 30% concentration, the reduction was 46.1% in relation to the control and coumarin. The concentration of 40% of the endocarp extract provided the lowest average in the mycelial growth of *C. gloeosporioides* (Table 5), with a reduction of 43.7% in relation to the control and 34.1% in relation to coumarin. This phytopathogen had its growth stimulated by the extract obtained from the leaves, at all concentrations tested, by the bark extract of the bark at a concentration of 20% and, by the extract from the seeds, at concentrations of 20%, 40% and 50% (Table 5).

*Fusarium* sp. lettuce isolate was the most sensitive fungus to the action of the leaf extract, obtaining the lowest averages of mycelial growth at concentrations of 30%, 40% and 50% (Table 5). The concentration of 40% caused a reduction of 4.4 cm in the diameter of the colonies, in relation to the control, and of 3.8 cm in relation to the coumarin. The extract obtained from the seeds also provided promising results for the *Fusarium* sp. obtained from cabbage, at a concentration of 10%, reducing the colony diameter by 59.5%, in relation to the control, and by 56.2% when compared to coumarin. The concentration of 40% of the seed extract caused a reduction of 18.5% and 17.5% in the mean diameter of the *S. rolfsii* colonies, in relation to the control and coumarin, respectively. This phytopathogen was the most resistant to the action of the extracts, in addition to having its mycelial growth stimulated, at concentrations of 10%, 40% and 50% of the leaves, and at all concentrations of the husks and endocarps (Table 5).

The extracts of *D. odorata* and *D. punctata* obtained from leaves, branches and seeds had no effect on the evaluated bacteria. However, extracts from the husks and endocarps of *D. odorata* and *D. punctata* inhibited the growth of *Escherichia coli* at the highest concentration tested. *D. punctata* husk extract also showed action against *Staphylococcus aureus*, at a concentration of 2000 µg·mL^−1^ (Table 6). The minimal bactericidal concentration (MBC) test indicated the bacteriostatic effect of the extracts.

## 3. Discussion

Regarding the yield results, data similar to those obtained in this study for cumaru seeds were found when comparing conventional and supercritical ethanolic extractions of bioactives, resulting in a yield of 46.5%, using Soxhlet apparatus [23]. The high yield values obtained by the Soxhlet extraction method were attributed to solvent recirculation and solute–solvent interactions [24]. The yield of extracts is fundamental in the cultivation and harvesting of medicinal plants, as it directly implies the fresh mass needed to obtain the products, the choice of the drying process, the reduction in cost estimates and less loss in the production chain of plants [25]. As for the presence of coumarin by TLC, it was observed in the extracts of the seeds of *D. odorata* and in all parts of the fruit of *D. punctata*, it has a molecular mass of 146.15 µg, melting point between 68 and 70 °C, boiling point of 303 °C and density of 0.94 g·cm^−3^ [26]. Coumarins are synthesized mainly in leaves, but occur at higher levels in fruits, followed by roots and stems; however, seasonal changes and environmental conditions can affect their occurrence in different parts of the plant [27].

According to the constituents identified in gas chromatography coupled to mass spectrometry (GC-MS), taraxasterol is present in the branches of *D. odorata* and in the leaves and branches of *D. punctata*. This constituent is a triterpenoid known to increase the inhibitory effects of anticancer drugs [28,29]. It also demonstrates a protective effect against rheumatoid arthritis, mediated by the modulation of inflammatory responses in mice [30], and has antimicrobial properties [31,32], which may have contributed to the antifungal action of extracts from these parts of the plant. In cumaru wood, the presence of isoflavone, retusin and several of their derivatives, odoratin and dipterixin, was observed in studies, while in the stem bark, in addition to isoflavone and odoratin, triterpenoids, such as lupeol and betulin, were also identified, and a mixture of fatty acids [33]. Among the constituents identified in the extracts of the husks and endocarps of the fruits of *D. odorata* and in the husks of the fruits of *D. punctata*, the sesquiterpenes stand out. These compounds were identified in greater numbers in the extract of the endocarps of *D. odorata* fruits, aromadendrene (9.2%), alpha-caryophyllene (3.9%), phenanthrene [3,2b]furan-4-methanol-1,2,3,4,4a,5,6,6a,7,11,11a,11b, dodecahydro-4,7,11-trimethyl (2.7%), beta-cubebene (2.3%), caryophyllene (1.6%), gama-cadinene (1.3%), gama-muurolene (1.0%), copaene (0.9%), and several biological activities have been reported for terpenoids, including antimicrobial activity [34,35], as was obtained for this extract in the control of *C. gloeosporioides* and *Fusarium* spp., with inhibitions greater than 50% being considered high antifungal activity [13]. The amount and chemical composition of plant oils and extracts are influenced by several factors, including the age of the plant, the type of tissue, the type of soil where the plant is grown and its habitat, climatic factors, times of material collection, and genetics [36,37]. In this sense, care during harvest, such as time of year, time of day, stage of development, choice of botanical part and material processing, should be taken into account so as not to influence the fungistatic or fungicidal potential of the substances [38].

Coumarin (1,2-benzopyrone) was confirmed by GC-MS in *D. odorata* seeds and in *D. punctata* husks, endocarps and seeds. The 3,4-dihydrocumarin found in the seeds of *D. punctata* was one of characteristics that differentiated the two plant species studied in this research. In addition, 30 to 40% of the dry weight of the cumaru seed consists of a light yellow, fragrant oil, which oxidizes rapidly in contact with air [39], which has numerous implications in folk medicine. The extract obtained from the seeds of *D. punctata* showed action against *Fusarium* spp. (kale), and the constituents 1,2-benzopyrone and 3,4-dihydrocoumarin present in this extract are part of the class of metabolites used in condiments, beverages, gelatins, puddings, perfumes and cosmetics, in addition to several coumarin derivatives that show antifungal activity [40].

As for the antifungal activity of the 1,2-benzopyrone standard, it was found that the concentration used was not the ideal one to reduce the mycelial growth of the phytopathogens used in the work; however, the extracts that have coumarin as a chemical constituent showed significant results. Fungi behave differently both in relation to the oil or extract used, and in relation to the lethal dilution or the dilution necessary to inhibit their growth [41]. Due to the contact of plants with pathogenic fungi, coumarins and lactones may have their synthesis increased. These substances, when released into the medium, can act as allelochemicals, inhibiting the action of predators, insects, microorganisms or weeds [42]. In addition, it is important to emphasize that the substances present in the composition of plant products can act synergistically and present broad fungicidal or fungistatic action [43,44]. In this sense, the results obtained by the husks and endocarps of the fruits, called residues, are positive when considering the future development of a product for application in the field in organic crops, where the use of pesticides for fungal control is not allowed. This material discarded by the producers after the processing of the seeds is also a possible source of reuse for the extraction of coumarin.

Regarding antibacterial activity, the classification for plant materials is based on the MIC, with strong inhibition up to 500 µg·mL^−1^, moderate inhibition between 600 and 1500 µg·mL^−1^, and weak inhibition above 1600 µg·mL^−1^ [45]. The results obtained for the extracts of the husks and endocarps of *D. odorata* and *D. punctata* fit the classification of weak inhibitors for the bacterium under test; however, these extracts present the largest number of compounds identified as sesquiterpenes, with their microbiological properties already reported, and in *D. punctata* extracts, 1,2-benzopyrone may also have positively influenced the action. A study of the antibacterial activity of coumarin and its derivatives against *Escherichia coli*, *Staphylococcus aureus*, *Bacillus cereus* and *Pseudomonas aeruginosa* showed that, especially for coumarin, all strains were susceptible [46]. Antibacterial studies with *Mikania glomerata* (guaco), a species whose chemical marker is coumarin, point to the existence of some degree of activity against Gram-positive and Gram-negative bacteria [47]. Important activities of this species were found against methicillin-resistant strains of *S. aureus* (MRSA) [48] and *E. coli* [49]. The results demonstrate that the antimicrobial activity of compounds from different plants and parts that compose them can be variable. However, the investigation of plant species with antimicrobial potential in plant species is absolutely relevant, in view of the increase in resistance of Gram-positive and negative bacteria to existing drugs, which makes health treatments difficult and increases mortality.

## 4. Materials and Methods

The specimens of *D. odorata* and *D. punctata* (Fabaceae) were found in the Tapajós unit of the Federal University of Western Pará (Ufopa), municipality of Santarém, PA, Brazil, under the following coordinates: 2°25′11″ S, 54°44′29″ W (*D. odorata*) and, 2°25′11″ S, 54°44′29″ W (*D. punctata*), and the altitude of 18 m. The specimens were deposited at the Ufopa Herbarium, under registration numbers HSTM 000556 (*D. odorata*) and HSTM 000,557 (*D. punctata*).

### 4.1. Collection and Extraction of Plant Materials

To obtain extracts from leaves, branches and fruits, the latter were separated into husk (epicarp plus mesocarp), endocarps and seeds of *D. odorata* and *D. punctata* and the materials were weighed, placed in paper bags or on trays and placed in an oven at 45 °C, with forced air circulation for 120 h. After this period, they were weighed again to obtain the dry mass and crushed for extraction.

For the ethanolic extractions, 40 g were weighed for the leaves, branches and endocarps, 22 g for fruit husks and 8 g for seeds. Procedures were performed in triplicate using Soxhlet apparatus, with P.A. 96% ethyl alcohol solvent and duration of 8 h for each procedure. The ethanolic solutions were concentrated in a rotary evaporator for 4 h at 45 °C to remove the solvent and obtain the extracts, which were stored in sterilized amber flasks and submitted to final drying in a desiccator for 24 h. Yields (%) were calculated by the following formula: (mass of extract (g)/mass of dry plant material (g)) × 100 [50].

### 4.2. Phytochemical Analysis of the Extracts

The 1,2-benzopyrone (simple coumarin) used as a standard in the antifungal assays was isolated from cold hexane extraction of cumaru seeds in triplicate. The extraction product was subjected to the crystallization process by adding hexane, heating this solution to 60 °C and volatilizing the solvent at room temperature. The impurities were removed by washing with hexane and vacuum filtration on a Buchner funnel, and coumarin crystals were obtained [50]. The purity of the isolated substance was verified by melting point, thin layer chromatography (TLC) and as chromatography coupled to mass spectrometry (GC-MS).

The presence of 1,2-benzopyrone in *D. odorata* and *D. punctata* extracts was qualitatively analyzed by TLC and GC-MS. In the TLC, aluminum plates (10 × 10 cm) and silica gel 60 with a fluorescent indicator and layer thickness of 0.20 mm were used. Then, 1 mL of homogenized ethyl alcohol was added to each weighed sample (20 mg), and 10 μL aliquots of their volumes were applied on the chromatographic plate, at distances of 1.5 cm each. The 1,2-benzopyrone standard was weighed at 10 mg, 1 mL of methyl alcohol was added and 10 μL aliquots of its volume were applied to the chromatographic plates [51].

For the analysis of 1,2-benzopyrone (coumarin) in samples of both plant species, the system consisted of plates eluted using a mixture containing ether-ethylic toluene (1:1). Subsequently, these plates were dried and developed with 5% potassium hydroxide (KOH). The retention factors (Rf) were calculated by the following formula: Rf = h/H, where h = height of the sample from the application point; H = maximum height of the mobile phase [50].

For GC-MS, we used the HP-6890 chromatograph, HP-5MS capillary column (30 m × 0.25 mm × 0.25 μm) and detector operating at 70 eV, with a linear scan in the range of 30 to 500 u.m.a. The chromatographic conditions were as follows: injector temperature 250 °C and detector temperature 300 °C, helium as carrier gas (1.0 mL·min^−1^); temperature programming 80 °C to 280 °C at a rate of 5 °C·min^−1^ and injection of 1 μL of coumarin and extracts. Compounds were identified by comparison with the NIST-11 library.

### 4.3. Antifungal Activity of the Extracts

The antifungal assays were carried out with the following phytopathogenic fungi: *Cercospora longissima* obtained from lettuce leaves (*Lactuca sativa*), *Colletotrichum gloeosporioides* isolated from pumpkin fruits (*Cucurbita pepo*), two *Fusarium* spp. isolates obtained from lettuce and cabbage leaves (*Brassica oleracea*) and *Sclerotium rolfsii* from sweet pepper (*Capsicum annuum*) fruits.

For each extract obtained from the different plant parts of *D. odorata* and *D. punctata*, a solution was prepared containing crude extract and sterilized distilled water, in the proportion of 1:1, with the addition of polyvinylpyrrolidone (PVP), at a concentration of 1:4 (extract: PVP). The solutions were placed in potato-dextrose-agar (PDA) culture medium, to obtain the following concentrations: 10%; 20%; 30%; 40% and 50% (*w*:*v*). The 1,2-benzopyrone standard was weighed (10 mg), diluted in 1 mL of methyl alcohol and an aliquot of 300 µL was removed from its volume for addition to the PDA medium (concentration of 1:1000).

After the addition and solidification of the extracts and the standard in the medium, a 0.4 cm disk containing the fungal structures was added centrally [52], which were incubated at 25 °C, under a photoperiod of 12 h. Fungi deposited only on BDA served as a control. The experimental design was completely randomized (DCR) in a factorial scheme (6 × 6 × 5) (six extracts, six concentrations, including the control (zero), and five phytopathogens), with four replications. The evaluations were carried out by measuring the average diameter of the colonies daily, until the control occupied the entire plate.

### 4.4. Antibacterial Activity of Extracts

The minimal inhibitory concentration (MIC) antibacterial assay was performed according to the recommendations of the M7-A6 protocol [53]. The microorganisms used were *Burkholderia cepacia* (ATCC 25416); *Escherichia coli* (ATCC 11775); *Pseudomonas aeruginosa* (ATCC 13388) and *Staphylococcus aureus* (ATCC 6538).

Next, 50 mg of all extracts obtained from *D. odorata* and *D. punctata* were weighed. Each sample was diluted in Mueller–Hinton (MH) culture medium to a concentration of 8 mg·mL^−1^, containing 10% DMSO (dimethylsulfoxide). This concentration corresponded to 2 mg·mL^−1^ in the first compartment of the ELISA microplate. From the standardized inoculum solutions, serial dilution was performed to obtain a concentration of 5 × 10^5^ CFU·mL^−1^ in the microplate wells. The concentrations of 2000; 1000; 500; 250; 125; 62.5; 31.25; 15.62; 7.81; 3.91 and 1.95 µg·mL^−1^ were evaluated. The antibiotic chloramphenicol (0.5 mg·mL^−1^) served as a control. Subsequently, 100 µL of the standardized inoculum was added to columns 2 to 12, and the plates were sealed with Parafilm.

The bacteria contained in the microplates were incubated in an oven at 36 ± 1 °C for 24 h, after which 50 µL of a 0.1% solution of 2,3,5-triphenyl-tetrazolium chloride (TTC) was deposited in all wells and re-incubated for a period of three hours. The MIC was defined as the lowest concentration of the samples capable of preventing the appearance of red staining, given to the medium when the cells present respiratory activity. After the MIC test, the minimum bactericidal concentration (MBC) test was performed, determining the bactericidal or bacteriostatic action of the extracts, depending on the bacterial appearance on the plates with MH.

### 4.5. Statistical Analysis

Analysis of variance (ANOVA) was performed with the extract yield data and the treatment means were compared using the Tukey test (*p* ≤ 0.05). With the antifungal activity data, ANOVA and the treatment means were compared using the Skott–Knott test (*p* ≤ 0.05). All statistical analyses were performed using the Assistat 7.7 beta software (Campina Grande, Brazil) [54].

## 5. Conclusions

In this comparative study between the species *Dipteryx odorata* and *Dipteryx punctata*, the presence of coumarin was verified in the seeds of both species, and for *D. punctata*, this substance was also found in the husk and endocarps of the fruits, adding value to this material that is discarded. The species under study showed low antimicrobial potential against bacteria, but showed promise in the use of its fruits for antifungal action. The extracts are being chemically analyzed to quantify the compounds, in addition to coumarin. The products and concentrations that showed promising results in the in vitro assays will be tested in harvested fruits and/or plants under nursery conditions, to confirm their potential. In the future, field research should be carried out to examine how these bioproducts will behave under these conditions so that they can be recommended.

## Figures and Tables

**Table 1:** Average yield of the ethanolic extracts obtained via Soxhlet of different parts of *Dipteryx odorata* and *Dipteryx punctata*.

Materials	Average Yield (%)	
	*Dipteryx odorata*	*Dipteryx punctata*
Leaves	26.36 b	20.87 bc
Branches	26.88 b	26.88 b
Husks	44.15 a	54.21 a
Endocarps	13.72 c	15.49 c
Seeds	48.89 a	44.52 a
CV (%) ^1^	10.94	12.26

^1^ Averages followed by the same letters in the columns do not differ among each other through the Tukey test (*p* ≤ 0.01). CV: coefficient of variation.

**Table 2:** Chemical constituents of ethanolic extracts of *Dipteryx odorata* characterized by gas chromatography coupled to mass spectrometry (GC-MS).

Identified Substances										
RT (min)	Leaves		Branches		Husks		Endocarps		Seeds	
	M	%	M	%	M	%	M	%	M	%
11.94	-	-	-	-	-	-	Copaene	0.9	-	-
12.17	-	-	-	-	-	-	-	-	148	2.3
12.30	-	-	-	-	-	-	189	1.1	-	-
13.00	-	-	-	-	189	1.4	Caryophyllene	1.6	-	-
13.51	-	-	-	-	-	-	-	-	2H-1-Benzopyran-2- one (coumarin)	87.3
13.81	-	-	-	-	Alpha-caryophyllene	4.0	Alpha-caryophyllene	3.9	-	-
14.32	-	-	-	-	-	-	Gama-muurolene	1.0	-	-
14.46	-	-	-	-	Germacrene D	1.5	Beta-cubebene	2.3	-	-
15.41	-	-	-	-	-	-	Gama-cadinene	1.3	-	-
16.71	-	-	205	5.0	-	-	-	-	-	-
16.72	-	-	-	-	Spathulenol	14.0	Aromadendrene	9.2	-	-
16.84	-	-	-	-	Caryophyllene oxide	4.6	205	2.5	-	-
17.43	-	-	-	-	220	7.5	220	4.0	-	-
17.73	-	-	-	-	-	-	-	-	182	10.4
18.06	-	-	-	-	-	-	220	1.0	-	-
18.42	-	-	-	-	220	1.8	220	1.6	-	-
19.84	-	-	129	2.7	-	-	220	0.7	-	-
19.86	151	10,4	-	-	-	-	-	-	-	-
19.90	179	2.4	-	-	-	-	-	-	-	-
19.98	182	1.4	-	-	-	-	-	-	-	-
20.23	-	-	-	-	238	1.5	-	-	-	-
20.45	180	4.8	-	-	-	-	-	-	-	-
20.84	-	-	-	-	218	2.4	-	-	-	-
22.30	-	-	-	-	-	-	234	2.1	-	-
22.31	-	-	-	-	234	3.2	-	-	-	-
22.73	-	-	-	-	220	1.7	220	1.5	-	-
23.67	-	-	-	-	218	1.8	218	1.2	-	-
24.23	-	-	-	-	-	-	218	1.2	-	-
24.29	-	-	-	-	236	2.0	-	-	-	-
34.39	-	-	-	-	Fenanthrene [3,2]furan-4- methanol-1,2,3,4,4a,5,6,6a,7,11,11a, 11b, dodecahydro-4,7,11-trimethyl	2.3	Fenanthrene [3,2]furan-4- methanol-1,2,3,4,4a,5,6,6a,7,11,11a, 11b, dodecahydro-4,7,11-trimethyl	2.7	-	-
36.31	-	-	-	-	341	2.0	331	1.4	-	-
37.52	-	-	-	-	374	2.8	374	3.5	-	-
38.15	-	-	-	-	-	-	429	1.3	-	-
39.60	-	-	405	16.7	-	-	-	-	-	-
39.62	-	-	-	-	429	21.4	-	-	-	-
39.63	-	-	-	-	-	-	400	31.6	-	-
40.00	-	-	-	-	402	5.0	-	-	-	-
40.01	-	-	-	-	-	-	429	6.9	-	-
41.33	-	-	-	-	-	-	405	2.4	-	-
41.70	-	-	-	-	400	6.0	-	-	-	-
41.71	-	-	-	-	-	-	429	7.3	-	-
42.58	-	-	405	11.7	-	-	-	-	-	-
42.77	-	-	-	-	429	5.1	429	5.8	-	-
48.75	424	15.7	-	-	-	-	-	-	-	-
49.38	426	65.3	-	-	-	-	-	-	-	-
49.39	-	-	Taraxas-terol	63.9	426	8.0	-	-	-	-
Total	100		100		100		100		100	

RT = retention time; M = mass spectrum; % = relative percentage.

**Table 3:** Chemical constituents of ethanolic extracts of *Dipteryx punctata* characterized by gas chromatography coupled to mass spectrometry (GC-MS).

Identified Substances										
RT (min)	Leaves		Branches		Husks		Endocarps		Seeds	
	M	%	M	%	M	%	M	%	M	%
12.16	-	-	-	-	-	-	-	-	2H-1-Benzopyran-2-one,3,4- dihydro (3,4-Dihydrocoumarin)	25.2
13.47	-	-	-	-	-	-	-	-	2H-1-Benzopyran-2- one (coumarin)	43.0
13.53	-	-	-	-	2H-1-Benzopyran-2-one (coumarin)	2.8	2H-1-Benzopyran-2-one (coumarin)	8.6	-	-
13.75	-	-	-	-	-	-	161	1.0	-	-
15.42	-	-	-	-	*Gama*-cadinene	1.2	-	-	-	-
16.39	-	-	-	-	235	1.5	-	-	-	-
16.72	-	-	-	-	-	-	205	1.1	-	-
16.73	-	-	-	-	Spathulenol	14.6	-	-	-	-
16.85	-	-	-	-	205	2.4	-	-	-	-
17.79	-	-	-	-	-	-	-	-	182	21.9
18.43	-	-	-	-	220	2.5	-	-	-	-
18.49	-	-	-	-	-	-	-	-	231	0.4
19.14	-	-	-	-	220	1.7	-	-	-	-
19.61	-	-	-	-	220	0.6	-	-	-	-
19.84	-	-	-	-	220	1.4	-	-	-	-
19.91	-	-	-	-	220	0.8	-	-	-	-
20.24	-	-	-	-	207	5.5	-	-	-	-
20.27	179	2.8	-	-	-	-	-	-	-	-
20.37	-	-	-	-	177	9.9	-	-	-	-
20.51	-	-	-	-	-	-	-	-	182	3.3
20.54	-	-	-	-	-	-	-	-	182	0.3
20.68	-	-	161	49.0	-	-	-	-	-	-
20.69	-	-	-	-	-	-	-	-	167	0.9
20.86	-	-	-	-	193	2.2	-	-	-	-
20.93	179	4.3	-	-	-	-	-	-	-	-
20.98	-	-	179	35.9	-	-	-	-	-	-
21.01	179	0.8	-	-	-	-	-	-	-	-
21.08	159	2.1	-	-	-	-	-	-	-	-
21.09	-	-	-	-	-	-	182	57.7	-	-
21.39	-	-	-	-	-	-	207	31.6	-	-
22.31	-	-	-	-	219	3.2	-	-	-	-
22.74	-	-	-	-	220	2.7	-	-	-	-
23.68	-	-	-	-	221	2.1	-	-	-	-
24.24	-	-	-	-	218	1.8	-	-	-	-
25.23	-	-	-	-	Ethylic ester of hexadecanoic acid	1.7	-	-	-	-
27.45	207	9.1	-	-	-	-	-	-	-	-
28.41	-	-	-	-	Ethyl ester of oleic acid (ethyl oleate)	9.4	-	-	-	-
28.52	-	-	-	-	281	1.4	-	-	-	-
31.32	-	-	-	-	310	1.7	-	-	-	-
31.33	-	-	-	-	-	-	-	-	310	2.2
32.29	-	-	-	-	-	-	-	-	259	2.8
32.30	-	-	-	-	Ester bis (2-ethyl-hexyl) of hexanodioic acid	2.7	-	-	-	-
32.92	-	-	-	-	300	4.1	-	-	-	-
34.41	-	-	-	-	Fenanthrene [3,2]furan-4-methanol-1,2,3, 4,4a,5,6,6a,7,11,11a, 11b, dodecahydro-4,7,11-trimethyl	5.7	-	-	-	-
34.53	-	-	-	-	298	1.5	-	-	-	-
38.50	-	-	-	-	361	4.4	-	-	-	-
39.64	-	-	-	-	429	3.7	-	-	-	-
40.01	-	-	-	-	360	1.9	-	-	-	-
40.27	-	-	-	-	429	2.0	-	-	-	-
41.00	-	-	-	-	405	2.9	-	-	-	-
48.76	551	29.0	-	-	-	-	-	-	-	-
49.39	Taraxas-terol	51.9	Taraxas-terol	15.1	-	-	-	-	-	-
Total	100		100		100		100		100	

RT = retention time; M = mass spectrum; % = relative percentage.

**Table 4:** Antifungal activity of the ethanolic extracts of *D. odorata*, in different concentrations.

		Mean Diameter of the Phytopathogen Colonies (cm)				
Extracts	Concentration (%)	*Cercospora longissima*	*Colletotrichum gloeosporioides*	*Fusarium* sp. (Lettuce)	*Fusarium* sp. (Kale)	*Sclerotium rolfsii*
Control	0	3.9 bD	4.8 cC	7.5 aB	7.9 aA	8.1 aA
Coumarin	0.1	3.9 bC	4.1 dC	6.9 bB	7.3 bB	8.0 aA
Leaves	10	3.5 cD	5.5 bC	7.4 aB	8.2 aA	8.3 aA
	20	3.4 cD	5.4 bC	7.5 aA	7.9 aA	7.0 bB
	30	2.7 eE	5.7 aD	7.5 aB	8.1 aA	6.8 bC
	40	3.0 dC	5.9 aB	6.1 cB	7.9 aA	7.9 aA
	50	3.1 dD	5.8 aC	5.8 cC	8.2 aA	7.7 aB
Branches	10	3.9 bD	4.2 dD	5.8 cC	7.3 bB	8.0 aA
	20	3.7 bD	4.1 dD	5.9 cC	7.6 aB	8.2 aA
	30	3.6 cE	4.1 dD	5.6 cC	7.1 bB	8.1 aA
	40	3.8 bD	4.1 dD	5.9 cC	6.8 cB	8.3 aA
	50	3.8 bD	4.1 dD	6.1 cC	6.8 cB	7.8 aA
Husks	10	4.9 aC	4.8 cC	8.0 aA	7.0 bB	8.2 aA
	20	5.3 aC	4.5 cD	6.6 bB	6.6 cB	8.3 aA
	30	5.2 aD	4.4 dE	7.8 aB	6.5 cC	8.3 aA
	40	5.2 aC	4.0 dD	6.4 bB	6.4 cB	8.3 aA
	50	4.8 aD	3.9 dE	6.6 bB	5.8 dC	8.3 aA
Endocarps	10	3.2 dC	3.5 eC	6.7 bB	6.7 cB	8.3 aA
	20	2.8 eC	2.9 fC	5.3 dB	5.3 eB	8.3 aA
	30	2.5 eC	2.4 gC	5.1 dB	4.7 fB	8.3 aA
	40	2.4 eC	1.7 hD	3.5 eB	3.5 gB	8.3 aA
	50	2.4 eC	2.1 hC	3.4 eB	3.2 gB	8.3 aA
Seeds	10	3.5 cD	5.5 bC	7.6 aA	6.0 dB	5.3 dC
	20	3.5 cE	5.4 bD	7.2 aB	8.1 aA	6.1 cC
	30	3.5 cC	5.2 bB	7.2 aA	7.2 bA	6.9 bA
	40	2.9 dD	5.3 bC	5.0 dC	6.1 dB	7.0 bA
	50	3.0 dD	4.9 bC	5.4 dB	5.8 dB	7.6 aA
CV (%)						5.31

Means followed by the same lowercase letters in the columns and the same uppercase letters in the rows do not differ from each other by the Skott–Knott test (*p* ≤ 0.05). CV: coefficient of variation.

**Table 5:** Antifungal activity of the ethanolic extracts of *D. punctata*, in different concentrations.

		Mean Diameter of the Phytopathogen Colonies (cm)				
Extracts	Concentrations (%)	*Cercospora* *longissima*	*Colletotrichum gloeosporioides*	*Fusarium* sp. (Lettuce)	*Fusarium* sp. (Kale)	*Sclerotium* *rolfsii*
Control	0	3.9 eD	4.8 cC	7.5 bB	7.9 aA	8.1 bA
Coumarin	0.1	3.9 eD	4.1 dD	6.9 cC	7.3 bB	8.0 bA
Leaves	10	3.1 fD	5.5 aC	7.4 bB	7.8 aB	8.3 aA
	20	3.1 fE	5.2 bD	7.3 bB	6.4 dC	8.1 bA
	30	2.9 fD	5.5 aB	4.2 fC	8.2 aA	8.1 bA
	40	3.2 fC	5.9 aB	3.1 gC	8.2 aA	8.3 aA
	50	2.7 fE	5.6 aC	4.0 fD	6.7 cB	8.3 aA
Branches	10	3.9 eD	4.1 dD	6.2 dC	7.4 bB	8.0 bA
	20	3.8 eD	4.1 dD	6.0 dC	7.5 bB	8.1 bA
	30	3.8 eD	3.9 dD	5.7 eC	6.8 cB	8.1 bA
	40	3.9 eD	4.2 dD	5.6 eC	6.6 dB	7.9 bA
	50	3.8 eD	4.0 dD	5.8 dC	6.5 dB	8.0 bA
Husks	10	5.6 bB	5.1 cC	8.2 aA	7.9 aA	8.3 aA
	20	5.5 bB	5.2 bB	8.3 aA	8.2 aA	8.3 aA
	30	6.1 aC	4.2 dD	7.3 bB	7.5 bB	8.3 aA
	40	5.5 bB	4.7 cC	8.2 aA	8.0 aA	8.3 aA
	50	5.2 cC	4.5 dD	7.3 bB	8.1 aA	8.3 aA
Endocarps	10	3.4 eC	4.2 dB	8.0 aA	8.0 aA	8.3 aA
	20	3.3 fE	4.1 dD	7.1 cC	7.5 bB	8.3 aA
	30	3.0 fD	3.9 dC	6.0 dB	6.4 dB	8.3 aA
	40	3.2 fC	2.7 eD	5.4 eB	5.6 eB	8.3 aA
	50	3.7 eE	4.0 dD	7.1 cC	7.6 bB	8.3 aA
Seeds	10	2.4 gE	4.9 cC	8.3 aA	3.2 hD	7.7 bB
	20	3.0 fE	5.5 aC	6.9 cB	5.1 fD	7.5 cA
	30	2.1 gD	5.0 cC	6.6 cB	6.4 dB	7.0 dA
	40	4.5 dC	5.5 aB	6.9 cA	7.0 cA	6.6 eA
	50	4.1 eD	5.3 bB	6.9 cA	4.5 gC	7.0 dA
CV (%)						4.3

Means followed by the same lowercase letters in the columns and the same uppercase letters in the rows do not differ from each other by the Skott–Knott test (*p* ≤ 0.05). CV: coefficient of variation.

**Table 6:** Minimum inhibitory and minimum bactericidal concentrations of ethanolic extracts of *Dipteryx odorata* and *Dipteryx punctata*.

Pathogenic Bacteria (µg·mL^−1^)	Gram-Negative						Gram-Positive	
	*Burkholderia* *cepacia* (ATCC 25416)		*Escherichia* *coli* (ATCC 11775)		*Pseudomonas* *aeruginosa* (ATCC 13388)		*Staphylococcus* *aureus* (ATCC 6538)	
*Dipteryx odorata*	MIC	MBC	MIC	MBC	MIC	MBC	MIC	MBC
Leaves	*	*	*	*	*	*	*	*
Branches	*	*	*	*	*	*	*	*
Husks	*	*	2000	*	*	*	*	*
Endocarps	*	*	2000	*	*	*	*	*
Seeds	*	*	*	*	*	*	*	*
*Dipteryx punctata*	MIC	MBC	MIC	MBC	MIC	MBC	MIC	MBC
Leaves	*	*	*	*	*	*	*	*
Branches	*	*	*	*	*	*	*	*
Husks	*	*	2000	*	*	*	2000	*
Endocarps	*	*	2000	*	*	*	*	*
Seeds	*	*	*	*	*	*	*	*

* Minimal inhibitory concentration > 2000µg·mL^−1^.

**Figure A1:**
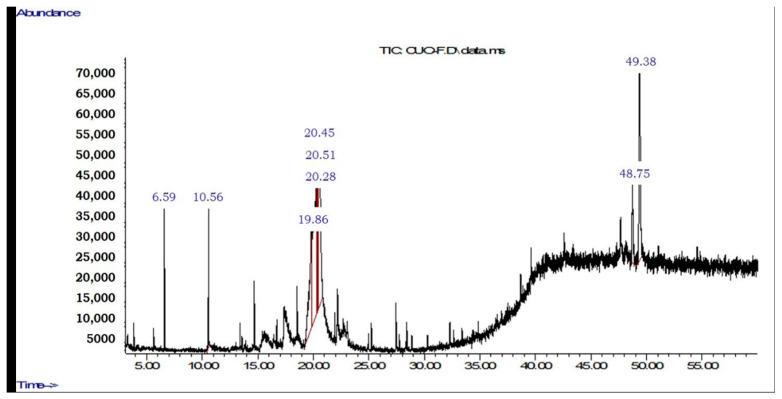
*Dipteryx odorata* leaves extract chromatogram.

**Figure A2:**
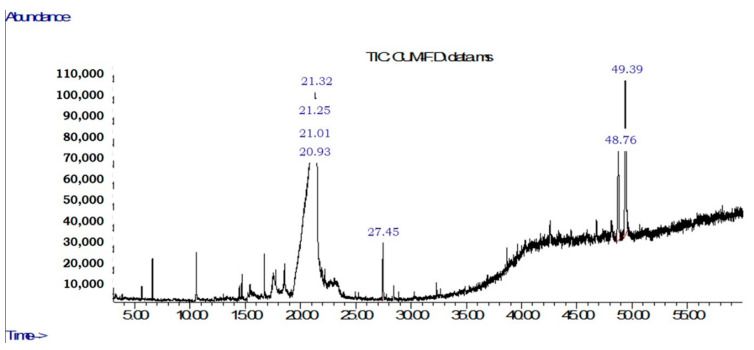
*Dipteryx punctata* leaves extract chromatogram.

**Figure A3:**
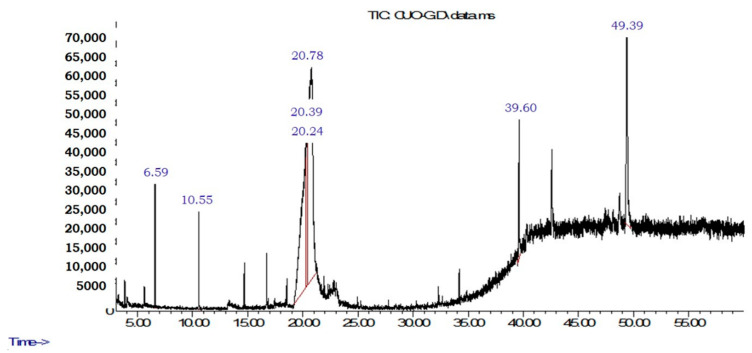
*Dipteryx odorata* branches extract chromatogram.

**Figure A4:**
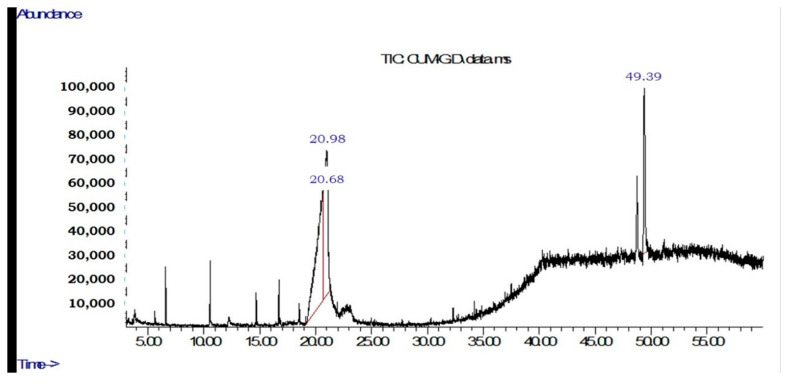
*Dipteryx punctata* branches extract chromatogram.

**Figure A5:**
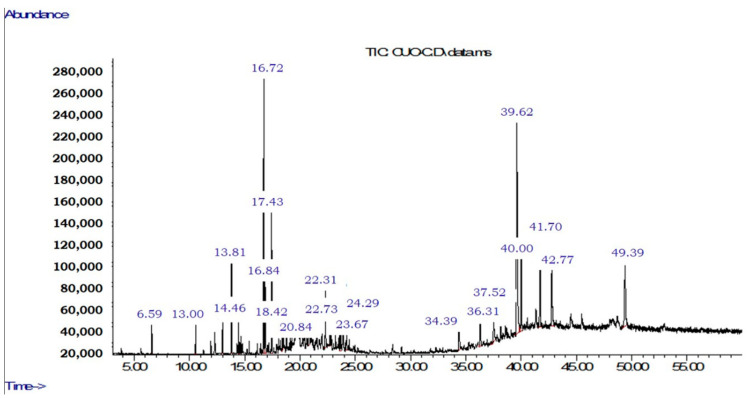
*Dipteryx odorata* husks extract chromatogram.

**Figure A6:**
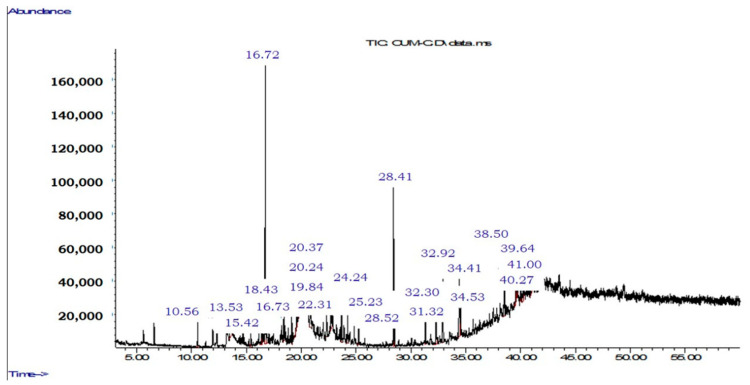
*Dipteryx punctata* husks extract chromatogram.

**Figure A7:**
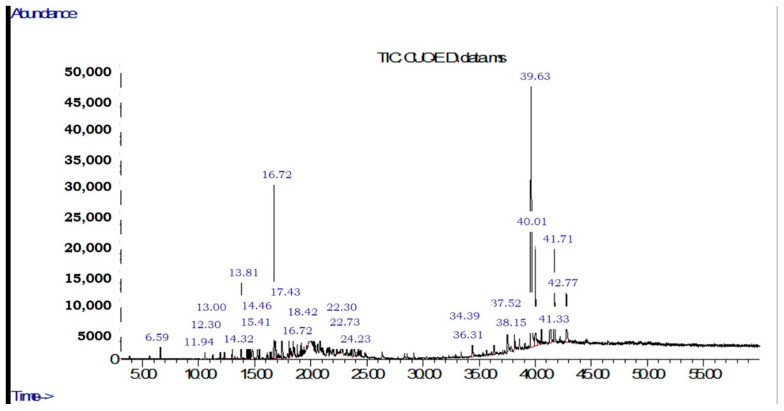
*Dipteryx odorata* endocarps extract chromatogram.

**Figure A8:**
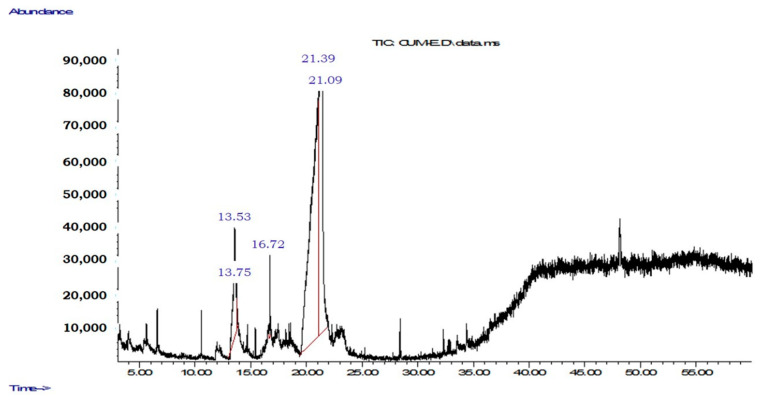
*Dipteryx punctata* endocarps extract chromatogram.

**Figure A9:**
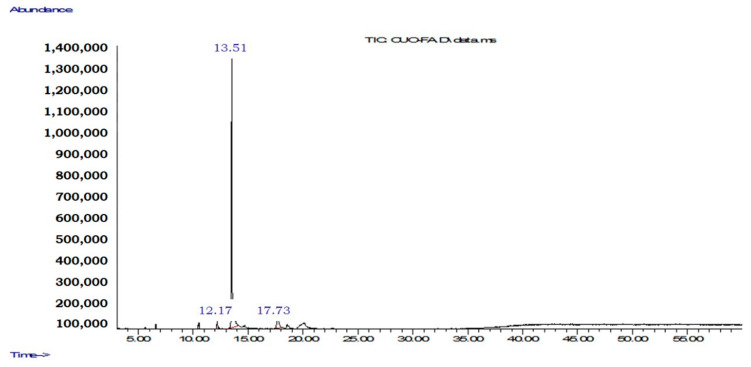
*Dipteryx odorata* seeds extract chromatogram.

**Figure A10:**
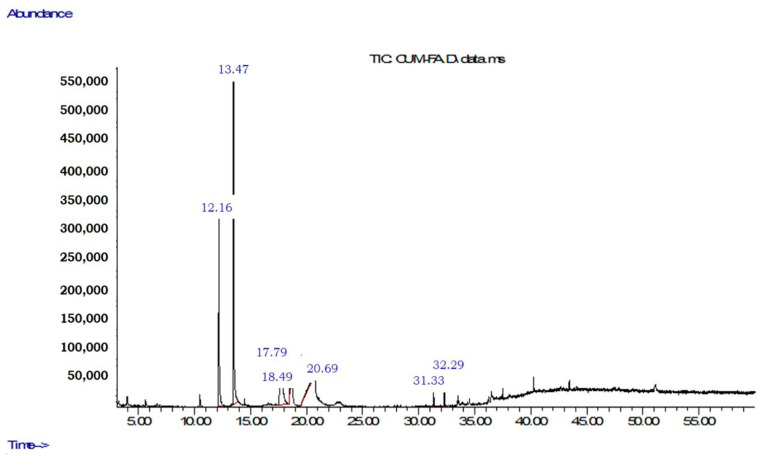
*Dipteryx punctata* seeds extract chromatogram.

## Data Availability

The data presented in this study are available in this article or its Appendix A.

## Sample Availability

Samples of ethanolic extracts from leaves, branches, husks, endocarps and seeds of *Dipteryx odorata* and *Dipteryx punctata* and isolated coumarin (1,2-benzopyrone) are available from the author B.C.M.d.S.
